# Experiences of Norwegian child and school health nurses with the “Starting Right™” child health assessment innovation: a qualitative interview study

**DOI:** 10.1186/s12913-022-08088-x

**Published:** 2022-06-01

**Authors:** Nastasja Robstad, Thomas Westergren, Eirin Mølland, Eirik Abildsnes, Kristin Haraldstad, Unni Mette Stamnes Köpp, Åshild Tellefsen Håland, Liv Fegran

**Affiliations:** 1 Department of Health and Nursing Science, Faculty of Health and Sports Sciences, University of Agder, P.O. Box 422, 4604 Kristiansand, Norway; 2 NORCE, Universitetsveien 19, 4630 Kristiansand, Norway; 3 Department of Economics and Finance, School of Business and Law, University of Agder, P.O. Box 422, 4604 Kristiansand, Norway; 4 Kristiansand municipality, P.O. Box 4, 4685 Nodeland, Norway; 5 Department of Psychosocial Health, University of Agder, P.O. Box 422, 4604 Kristiansand, Norway; 6 Department of Pediatrics, Sørlandet Hospital, P.O. Box 416, Lundsiden, 4604 Kristiansand, Norway

**Keywords:** Child and school health nurses, Child health, Digital innovation, Health service innovation, Public health nurses, School health

## Abstract

**Background:**

Although child health services are well established in Norway, the use of information technology for the systematic collection of evidence-based child- and proxy-reported health measures may be beneficial in the early identification of child development problems. The Norwegian “Starting Right™” health service innovation consists of parent- and child-reported online structured health assessments tools, including practical routines for child and school health assessments. The aim of this study was to explore the experiences of child and school health nurses with the Starting Right innovation.

**Methods:**

We used a qualitative design and conducted three focus group interviews with 18 child and school health nurses from three child health centres one year after the implementation of the innovation.

**Results:**

The experiences of professionals with the Starting Right innovation were captured by three themes: (1) the digital innovation could be used to obtain a good overview of a child’s health and development; (2) interpreting the questionnaires was a challenge; and (3) implementing the new digital innovation was time-consuming.

**Conclusions:**

Overall, the child and school health nurses experienced that the Starting Right innovation was useful for providing a comprehensive overview of child development and health. The challenges related to interpreting the parents’ scores and follow-up of children, as well as providing the questionnaires in relevant foreign languages, should be addressed to allow all children and families to be reached.

## Background

Although child health services in Nordic countries and Scotland are well developed for safeguarding the health and development of children, assessments and surveillance are hindered by the lack of evidence-based screening and evaluations of interventions [1]. In western countries screening of children’s health is recommended, but delivery and content vary considerably between countries [2]. According to Alexander [3], service providers in the United States (US) rarely rely on relevant clinical end-points to assess the intended and/or expected health changes in children which is similarly reported for the Nordic countries [1].

The proportion of children with poor health is growing slightly [4], and the trajectories of poor health commence and are identifiable early in the lives of children [5]. Shonkoff et al. [6] have suggested the need for initiatives to strengthen the resilience of children and efforts to protect children from unhealthy development. These initiatives should rely on evidence-based measures and interventions that are acceptable, feasible, and affordable, and they should strengthen informed professional and parental engagement [6].

In a study of the competence needs of child and school health nurses (CSHNs) in Finland, where child health care is comparable with that in Norway, Putkuri et al. [7] reported that intuitive and interpersonal competencies are well developed in child health care. However, it is necessary to advance and develop theoretical and evidence-based competencies, including the use and interpretation of health assessment scales. Furthermore, advanced electronic patient records and digital tools are required to support evidence-based screening tools and intervention strategies. For example, another study conducted in Finland using online tools determined the utility and appropriateness of a mental health screening tool (strength and difficulties questionnaire, SDQ), as well as how to overcome barriers to support parents and develop competence among CSHNs [8]. In a Swedish project nurses preferred using SDQ to assess mental health of children in child health clinics, but emphasized the importance of reducing individual and organizational level barriers [9]. In addition, systematically collecting data on the development and health of children can facilitate the distribution of services to geographical areas with the highest need for follow-up [10].

Nurses throughout the world have reported a need to establish positive therapeutic relationships with families amid growing concerns about child health challenges [11, 12], and it is necessary to validate their experience-based and situated concerns about children [12]. In particular, structured health assessments can improve the identification of children’s problems [13], provide decision support [14], create opportunities for evaluating interventions [15], and enhance communication [16]. Client-reported questionnaires concerning their health can enhance routine management by promoting client–clinician communication and client involvement [17]. Moreover, digital health assessments may be preferable and more acceptable for clients than paper-based questionnaires because of their faster completion time, lower cost, and increased response rate with resulting higher data quality [18]. However, the challenges associated with client-generated health data acquired using electronic tools include financial investment, privacy protection, and the exclusion of populations not familiar with electronic devices and platforms [18]. In addition, the ability to respond to patient generated health data could be challenging, including integration in clinical practices and processes [19]. In general, health care professionals are reported to have high digital literacy, but one-fifth report anxiety using information technology, which could endanger patient safety and the quality of service, and thus targeted training is required for staff with low digital literacy [20].

Validated structured health assessments using information technology need to be implemented in child and school health services. The Norwegian “Starting Right™” project uses digital proxy- and child-reported questionnaires to support CHSNs in assessments of the general and socio-emotional development of children’s health and development. This project has been piloted in routine child and school health care [21] using an online solution delivered by CheckWare Ltd. The Starting Right child and school health service innovation consists of parent- and child-reported online structured health assessments tools, including practical routines for use in assessments of child and school health services among children aged from six months to 16 years [21]. Previously validated questionnaires are used in the innovation, such as the SDQ [22], health-related quality of life (KIDSCREEN-27) [23, 24], general development (ages and stages questionnaire) [25], socio-emotional development (ages and stages questionnaire: social–emotional) [26], and anxiety (Spence child anxiety scale, short) questionnaires [27]. Parental acceptability and adoption by CSHNs were satisfactory in the pilot phase [21]. Before implementation, the preparation stage included monthly meetings with two CSHNs, having an official mandate for professional quality improvement in a large municipality in Norway. The instruments and starting age for screening were selected in collaboration with the relevant services, and information about the innovation was attached to an appointment letter sent to parents. Educational sessions concerning the questionnaires (5 h) and online tool (3 h) were provided to all CSHNs before implementation at the centres involved [21]. In the pilot phase, a medical secretary supported the CSHNs in distributing health assessments to parents prior to scheduled appointments, which increased the rate of adoption by CSHNs [21].

It is recommended to evaluate clinical experiences to identify challenges and required improvements, and to accommodate and provide further support with implementation [28]. In their design and evaluation implementation framework, Fixsen et al. [29] described seven core implementation components: (1) staff selection; (2) pre-service and in-service training; (3) ongoing coaching and consultation; (4) staff evaluation; (5) decision support data systems; (6) facilitative administrative support; and (7) systems interventions [29]. In the present study, we focused on the core component of staff evaluation with the specific aim of exploring the experiences of CSHNs with the Starting Right innovation.

## Methods

We employed a qualitative approach to explore the experiences of CSHNs with the implementation of the Starting Right innovation, which were assessed by conducting focus groups and then analysed the data by thematic analysis [30].

### Participants and setting

A purposive sampling of CSHNs was performed from three different child health centres in two municipalities in Southern Norway covering birth cohorts of approximately 450 children. The participants recruited by the manager at each centre comprised CSHNs with experience of working with the Starting Right child and school health service innovation since the start between October and December 2019. Eighteen CSHNs agreed to participate. CSHNs are the main professional group in the child and school health services. The CSHNs are required to have a minimum of one year of education (60 European Credit Transfer and Accumulation System (ECTS)) in addition to a bachelor’s degree in nursing. Norwegian CSHNs are specialists [31] and has a central role in the health promoting and preventive work in the municipalities [32]. The nurses are qualified to promote mental and physical health, promote good social and environmental conditions, prevent illness and injuries, and prevent and detect violence, abuse and neglect [32].

### Context

Norwegian child and school health services are organised in an interdisciplinary manner within the municipality primary health care system [1, 33]. The child health service in Norway is provided free of charge and it offers a minimum of 14 consultations for children aged 0 − 5 years located in the municipality of the child and parents, and the school health services provide individual appointments in at least grades 1 and 8 [34]. These services aim to promote the physical and mental health of children as well as their social conditions, and help and advice are offered by CSHNs, physicians, and physiotherapists [34].

### Data collection

Semi-structured focus groups were conducted to allow participants to discuss, reflect on, and exchange viewpoints, and this approach was found to be appropriate for meeting the aims of the present study [35]. Data were collected in suitable quiet rooms in the child health centres over a period of two months in November and December 2020. The focus groups were conducted for the CSHNs in three groups comprising five, six, and seven participants from each of the public health centres. The focus groups were moderated by two of the authors (LF and NR), where one acted as moderator and the other as co-moderator [36]. Both researchers are registered nurses with extensive experience in qualitative research studies; they were not directly involved in the implementation of the innovation, and they had no clinical experience or education in public health nursing.

The focus group questions were designed to obtain an understanding of the experiences of the CSHNs with the Starting Right innovation. The focus group guide covered aspects of the experiences of the CSHNs with using the digital tool (access to the software, electronic documentation, use of time, and extracting reports). In addition, the guide covered aspects concerning how the use of systematically collected data may contribute to the follow-up of children at the public health centres. The focus groups were audio recorded and lasted 47–58 min.

### Analysis

Thematic analysis was conducted with a combination of inductive and deductive data coding and analysis [30]. The interviews were manually transcribed verbatim by a professional transcriber and the analysis was conducted by the two researchers who moderated the focus groups. The transcribed text was imported into the data management software program NVivo 12 Pro for Windows [37]. The first phase of the analysis involved familiarisation with the data. All of the transcribed text was read and re-read to understand the meaning of the text as a whole. According to Braun and Clarke (page 87) [30], reading should be conducted in an “active way, searching for patterns and meanings”. During the second phase, initial codes were inductively generated by dividing the text into units based on the semantic level of meaning. In the third phase, the meaning units that shared the same features were clustered into three latent themes across the data set guided by the following overall themes from the interview guide: user friendliness, serviceability, and follow-up. In phases four and five, the themes were reviewed against the meaning units, defined, and named [30].

## Results

Eighteen female CSHNs aged 36–63 years participated in the study. They had 13–36 years of experience as registered nurses, with 2–26 years of experience as trained CSHNs. Thematic analysis was conducted according to three themes: (1) the digital innovation could be used as a method to obtain a good overview of a child’s health and development; (2) interpreting the questionnaires was a challenge; and (3) implementation of the new digital innovation was time-consuming. We now discuss these three themes in turn and illustrate them with quotes from the participants.

### The digital innovation could be used as a method to obtain a good overview of a child’s health and development

Despite the delay of the innovation due to the coronavirus outbreak, the overall response from the CSHNs concerning its serviceability was positive. They emphasised the importance of their involvement during the implementation process and using the innovation because they were aware of any bottlenecks or other issues, and the new routines influenced their daily work.

*It is about which tools we use… it is like telling a carpenter that you should use that hammer instead of the other hammer… if you take it to extremes. Of course, you could discuss the tools [questionnaires] that we use today because even if we had used them for years, other tools could be more suitable. But I think it [the decision about which tools to use] should come from us as users. (Focus group 1)*

They experienced that the systematic collection of data provided a more integrated and comprehensive impression of a child’s development and health.

*I think that we know about these issues but you understand them in a much more systematic manner when you have the possibility to score the child, you see? It is a little bit more than an overview… less speculation compared with what the parents have answered in detail [in the questionnaires] concerning emotions, for example. (Focus group 3)*

Although the majority of the CSHNs perceived the innovation as an important and useful tool for following up children, those with longer work experience found it less useful compared with those with shorter work experience.

*As I told you, I really don’t know if I am doing a better four-year consultation or school start consultation since we started this [innovation]. (Focus group 1)*

One important issue discussed in the focus group was that both the CSHNs and parents were more prepared when scoring their child’s strengths and weaknesses.

*I usually tell the parents who have filled out the questionnaires, that it is very helpful for me to read their answers in advance because I might not know their child. Because it gives you an impression of who this child is and you might then recognise some of those things, and then you can have a good conversation about it. (Focus group 2)*

The innovation was experienced as a tool that could be used for communicating information about deviations in child health and development to other services, such as kindergarten or family centres. However, the CSHNs wanted guidance about how to follow up on these deviations.

*There is no point in discovering these things if we don’t do anything different anyway. So, I think there should be a package of interventions… yes, something that tells us who we should contact or what we should do. If not, it will be a bit pointless I think. (Focus group 2)*

The CSHNs also discussed the possibility of involving other professionals such as teachers from the kindergarten to obtain a more nuanced picture of a child’s development and health.

However, the inability to merge data from the intervention with the patient’s journal software was described as an obstacle.

### Interpreting the questionnaires was a challenge

Providing ongoing and timely information to parents about how to fill in the questionnaires was experienced as crucial. According to the CSHNs, some of the parents might not have visited the public health centre for a long time, and they suddenly received information about the project and a request to respond to the questionnaires. The CSHNs suggested that contacting the parents in advance to prepare them for the upcoming questionnaire could have been beneficial.

*Yes, we should have contacted the parents and explained it to them because then we could have clarified issues that they were unclear about… they would have been able to ask questions instead of just receiving an information sheet to sign. (Focus group 2)*

They agreed that most parents actually wanted to contribute but the effort of filling in the questionnaires could have outweighed the benefits of participating.

*Do they understand the benefit of it? Because if they don’t, they probably won’t respond to the questions. They need to feel that it is beneficial to them [and their child], otherwise they will lack the motivation to participate in the project. (Focus group 2)*

The nurses were sometimes unsure whether the parents had actually understood the questions, which could be especially challenging for parents who were not native speakers of Norwegian.

*I think about the language… if they don’t understand the nuances and the words used in the questionnaires, then it could be difficult for them to understand. And it is important that they can read it in their own language. So, in fact, it is not strange that they don’t answer because they don’t understand it [the question], and this makes it difficult for them to answer. (Focus group 2)*

Another topic discussed in the focus groups was the relevance of the answers provided by the parents. One of the participants called the data collected a “fresh product” that could soon be outdated.

*One advantage is that we receive this information quickly. Right? If they tick that the child is often agitated with reduced concentration, becomes angry, and things like that, then immediately you can start to talk about it and say I can see that you have marked this out… do you want to talk about it… is it something that influences your everyday life? And if you run out of time, you can suggest another consultation to talk more about it. That provides us with an opportunity. (Focus group 3)*

The usefulness of the selected questionnaires was an issue discussed by the participants. Some considered that the questions were somewhat negatively biased by focusing on problems rather than a child’s strengths, but others considered that this was necessary to capture a comprehensive understanding of the child’s condition.

*We have more of a health promotion focus… they [the questions] are slightly more problem focused, like “do you often fall out with somebody”? But you must be a little problem focused as well to identify the difficulties [and other participants agreed]. If they [the questions] are just positively biased, we do not get the answers we need. (Focus group 3)*

Another issue that interested the participants was which parents responded and whether they helped the parents who needed extra support.

*Because we have talked about the fact that those who we really would like to answer the questionnaires before consultations, they don’t… those are the ones we would like to reach. But the resourceful ones, they usually fill in the forms and are happy with that. So, we wonder, have we got any further? (Focus group 1)*

Mothers generally responded to the questionnaires. The nurses suggested that this could be explained by various reasons, such as whether it was clear that both parents could answer the questionnaires, or if contact information was available for both mothers and fathers in the child’s journal. When both parents responded and evaluated their child’s development differently, this discrepancy could sometimes challenge the CSHN’s perception of the child.

*I have seen both [similar and different descriptions]. There are nuances in how mothers and fathers describe their child, and that is always interesting. (Focus group 3)*

Consultations with ethnic minority parents were described as even more challenging due to language and cultural issues.

*I have met quite a few minority families who don’t understand how they should respond to it [the questionnaire]… and then they do not understand, and haven’t filled it out… and how should I handle this, and I have to admit that I don’t do anything… I think it is a lot of fuss. (Focus group 2)*

Another challenging issue during follow-up was how some parents perceived and scored their child, and the different perception of the CSHN. This issue was a new experience for the participants that arose after implementing the innovation.

*I also have a mum who basically has quite a few challenges with her child who was followed up by the rehabilitation services… it was a completely normal score. That is how she perceived her child. So, it’s interesting. I thought maybe it would have been a higher score on the SDQ, but it was not. Maybe it’s about how you perceive your own child. (Focus group 1)*

In addition to differences in how parents perceived their child, the participants in the focus groups discussed parents who downplayed their child’s health issues to CSHNs.

*Because I also think that there can be a bit of under-reporting, and I also think that those (parents) who do not respond, these are often parents that may struggle with their child in one way or another, and who find it difficult, or may not know their children well enough. So, it will also be exciting to see this in the long run and how it will be. (Focus group 2)*

### Implementation of the new digital innovation was time-consuming

One of the main issues discussed by the CSHNs during the interviews was that using the new digital tool was perceived as more time-consuming, thereby resulting in extra work for both CSHNs and parents. The agenda at the child and school health centre was rather packed, with little or no spare capacity. Thus, finding time for the extra work during 6–10 consultations in one day could be challenging, especially if they had to combine their consultations with the doctors’ appointments (Focus group 3). However, the experiences of the CSHNs varied. Some described how prioritising the innovation replaced other topics that were previously included in the consultations. Others stated that the only difference was that documentation was conducted using the new digital tool instead of the electronic patient journal. Some initial challenges regarding the recording of the results were discussed in all of the interviews.

*And then we need some time to become confident ourselves to understand the reports and how to discuss the findings with parents. I really think it works mostly. But as we say, I had a negative attitude to logging results in the system because it is more time-consuming and you need more keystrokes than before. But when you have recorded the results and look at them [the completed questionnaire], I think it is good. (Focus group 2)*

After the CSHNs became acquainted with interpreting the results, they were still concerned about the amount of extra work required of the parents filling out (Focus group 1) and understanding multiple questionnaires.

*They take a lot of time just to record the results, just as we do. Then they must read through all the material, and then there are things they don’t understand… after having looked at the huge amount of text, they just put it off… and then they don’t bother to do anything about it… many parents have told me that that they looked at it, but they did not do anything about it. (Focus group 2)*

## Discussion

The aim of the present study was to explore the experiences of CSHNs with the implementation of the Starting Right child and school health service innovation. Overall, our findings indicated that the nurses were positive about the innovation. However, their experiences with the implementation varied and some of them were contradictory. Despite this motivation to implement the health service innovation, previous studies have shown that the implementation of structured self-reported health assessments can lead to diverse experiences, such as challenges including assessments as standard practice [38], given that the implementation process involves complex interactions between various factors [39]. Our findings are in agreement with previous studies that highlight, for example, scepticism regarding the validity of patient self-reporting, uncertain clinical benefits, concerns about workflow, and the lack of time [40, 41].

### The digital innovation could be used as a method to obtain a good overview of a child’s health and development

The CSHNs emphasised the importance of their involvement in the implementation and use of the innovation. Some CSHNs were involved in planning the innovation and in selecting the instruments used [21] but the participants in the present study considered that the currently used questionnaires should be changed according to their needs. Their practice was not based on traditions of using structured and validated health assessment tools, standardised forms, or systematic assessments in accordance with the World Health Organisation criteria for these tools [1], but they had to follow quite detailed national guidelines [33]. Thus, there could be differences in how to interpret their professional obligations in relation to the implemented assessments, and reciprocal and continuous adjustments of the innovation may be important beyond the pilot phase, as we reported previously [21]. Waldron et al. [26] also emphasised the need for continuing education when implementing routine outcome measures. Moreover, competence needs concerning the assessment rating scales and aetiology of symptoms, as well as appropriate interventions, were highlighted by Putkuri et al. [7] for a comparable child and school health service innovation in Finland.

 In the present study, the CSHNs found that using the innovation led parents to reflect more actively on their child’s development and health before the consultation. This finding appears to be consistent with previous investigations of the experiences of patients using self-reports of health, and thus these questionnaires may prompt patients to reflect on their own health [42]. Reflecting by using structured self-reported health assessments may increase the likelihood of raising personal issues with clinicians [42]. Furthermore, this may allow CSHNs to be more prepared for the consultation.

### Interpreting the questionnaires was a challenge

One of the main concerns raised by the CSHNs was the ability of parents to fill in the questionnaires. They found that some of the parents had problems understanding the meanings of the questions and they were concerned whether this might result in the parents postponing filling in the questionnaires or not responding. In addition, the issue of a language barrier, especially for ethnic minority parents, was considered a further problem that affected the ability of parents to respond. Several questionnaires have been translated into different languages and they were accessible as paper versions for translation support in the centres but online digital questionnaires were only accessible in Norwegian during the implementation phase of the Starting Right innovation. Clearly, the lack of access to questionnaires in different languages could have prevented ethnic minority parents from completing the questionnaires, as shown in previous studies [43, 44]. In Norway, approximately 15% of the population are immigrants [45], and in 2020, approximately 26.000 of the population received Norwegian language training of which 1 out of 4 where from Syria [46].

Another challenge to the implementation process may have been related to the “digital divide”, which is considered one of the major disadvantages of online structured self-reporting of health. A systematic review by Meirte et al. [18] found that people with limited access to computers or those who are generally computer illiterate are likely to be disadvantaged. If the parents encountered challenges because they lacked access to computers or mobile phones or had problems understanding the questionnaire, then they clearly would have had difficulty in filling in the questionnaires. Our previously reported parental response rate of 80–85% [21] also indicates that a group of parents did not respond to the questionnaires. These findings correspond with a Swedish study who revealed that nurses experienced that not all parents filled in the questionnaires [47]. The occasional parents who did not respond could be a challenge for the CSHNs in their daily clinical work regardless of their reasons for not responding.

 Some of the CSHNs experienced differences between their own perceptions of a child and the scores provided by some of the parents. They described cases of parents who both over- and under-estimated their child’s development and health challenges compared with their own perceptions. It is well known that people tend to respond in a manner that might be considered socially acceptable, especially when dealing with sensitive issues [48]. Social desirability can result in under-reporting due to the tendency to present oneself in a manner that is socially acceptable, or even to avoid criticism [49]. For some parents, social desirability might be an issue when scoring their child because they may be unwilling to reveal certain information. Some parents might also believe that questionnaires do not capture their concerns [50]. However, some of the nurses experienced the opposite where parents reported concerns about their children that were not perceived by the CSHNs. These findings agree with those obtained in other studies where low agreement was observed between the assessments of health professionals and parents regarding their children [51, 52]. Previous studies have also highlighted the need for clear cut-off values and interpretations of questionnaires such as the SDQ in different situations and contexts [53], and it may be necessary to use population-level data to improve these interpretations. On the other hand, the scores obtained from questionnaires might provide health professionals with information regarding previously unidentified issues [54], thereby allowing them to provide appropriate advice or interventions to meet the needs of children [55]. Several nurses argued that although most of the information regarding children was known to them, the specific conditions of children were clarified by the questionnaires, as also shown previously [56]. An integrative review by Lines et al. [12] highlighted that working with vulnerable children involves putting “pieces of a jigsaw puzzle” together through communication and validation. In the present study, the discrepancies observed by CSHNs between different information sources suggest that health assessments alone may not be sufficient to meet the needs of CSHNs. Lines et al. [12] argue that nurses have concerns about making mistakes and harming children or families when trying to provide support, or take action based on their “gut feeling” that something is wrong. We will argue that different information sources, including questionnaire-based health assessment scores, are needed to support nurses in taking appropriate action. Thus, although questionnaires can be considered tools for formally assessing the health situations of children, they do not entirely solve the dilemma of balancing surveillance with support. They cannot necessarily guide a quick and obvious response, which may have been required by nurses in the present study, as well as in general [12]. The systematic mapping of a child’s health and development may result in an increased commitment to the further utilisation of knowledge, and the nurses suggested that there is a requirement for a framework to determine how and when follow-ups should be performed for children with special needs. These findings echo those obtained in a systematic review by Boyce et al. [41] who reported that one of the barriers that hinders the implementation of questionnaire-based health assessments may be uncertainty about how the scores should be interpreted and how to transfer these interpretations into clinical care. Greenhalgh et al. [57] note that clinicians often have limited knowledge about how to implement structured questionnaire-based data in clinical practice. Similarly, Putkuri et al. [7] found that CSHNs need greater competence in using assessment rating scales, in understanding the aetiology of mental health symptoms in children, and of knowledge of the chain of care as well as preventive interventions.

The CSHNs were very concerned about whether the intervention might enhance the identification and support of families and children with health care issues. A realist synthesis by Greenhalgh et al. [42] showed that the use of questionnaire-based health assessments by clinicians is strongly influenced by their professional roles and relationships with patients. Clinicians indicated that standardised patient reported outcome measures (PROMs) could hinder communication and relationships with patients, whereas more individualised PROMs could strengthen the dialogue [42].

### Implementation of the new digital innovation was time-consuming

The time required by the innovation was considered an important issue by CSHNs and they were concerned about how it influenced their current working situation. In particular, they were concerned about the time taken to use the innovation during the implementation phase, such as when distributing questionnaires and access reports in the system. Implementing questionnaire-based health assessments is time-consuming [43] and although online tools can facilitate more time-efficient assessments, Meirte et al. [18] reported that time constraints remain barriers that may be related to digital literacy. The nurses described the value of access to support, especially concerning technical issues during the implementation phase, which may reflect challenges concerning digital literacy among CSHNs and that special support and training could be needed, as reported previously [20]. The issues of digital literacy and level of digital support could also explain the difference in the adoption rate identified in a pilot study of the implementation of the Starting Right innovation [21], in which the adoption rate was 96% in a group supported by a medical secretary in the pilot phase, but only 55% among CSHNs with no support. This discrepancy may indicate that CSHNs need extended support to overcome barriers related to time requirements when implementing the innovation.

### Strengths and limitations

The present study was conducted during the COVID-19 pandemic. As a consequence, some of the child and school health services temporary suspended their implementation or use of the Starting Right project, which inevitably affected the experiences of the nurses with the innovation. Nevertheless, we successfully recruited 18 CSHNs with a wide range of ages and years working as CSHNs, and thus a broad range of experience in public health nursing; these factors can be considered strengths of this study. Another strength was that the focus groups were moderated by two researchers who were registered nurses, and part of the research group, but they were not involved in the implementation of the innovation.

Although the Starting Right project had only been piloted for about one year, abundant data were obtained from the initial phase of the implementation. However, if the interviews had been conducted later, we could have obtained information about how the questionnaires were used after the initial challenges were addressed. In addition, our participants only comprised female nurses. Including male participants in Norwegian studies is a challenge because the proportion of male CSHNs in Norway in 2018 was only 0.3% [58].

## Conclusions

Overall, the CSHNs considered that the Starting Right innovation for child development and health assessments was useful for providing a comprehensive overview of the development and health of children. However, several challenges were identified, especially regarding how to interpret the scores provided by parents and how to use these scores to follow up their children. There is also a need to provide online questionnaires in different languages to reach all children and families.

## Data Availability

All relevant data are presented in this paper. Transcripts from the study are available from the corresponding author on reasonable request.

## Abbreviations

CSHN: child and school health nurse
ECTS: European Credit Transfer and Accumulation System

## Declarations

All participants gave written informed consent to participate in the study, and were informed about their rights according to data protection regulations [59].
